# Supplementary material for: The interactome of intact mitochondria by cross-linking mass spectrometry provides evidence for coexisting respiratory supercomplexes
Source: Mol Cell Proteomics. 2017 Dec 8;17(2):216–32. doi: 10.1074/mcp.RA117.000470 (PMC5795388; doi:10.1074/mcp.RA117.000470)
Supplement: Supplemental Data [file supp_RA117.000470_133922_0_supp_23978_fzffwf.zip › spectra_annotation/mito_spectra_annotation/278-1-3-1-7-1.pdf]

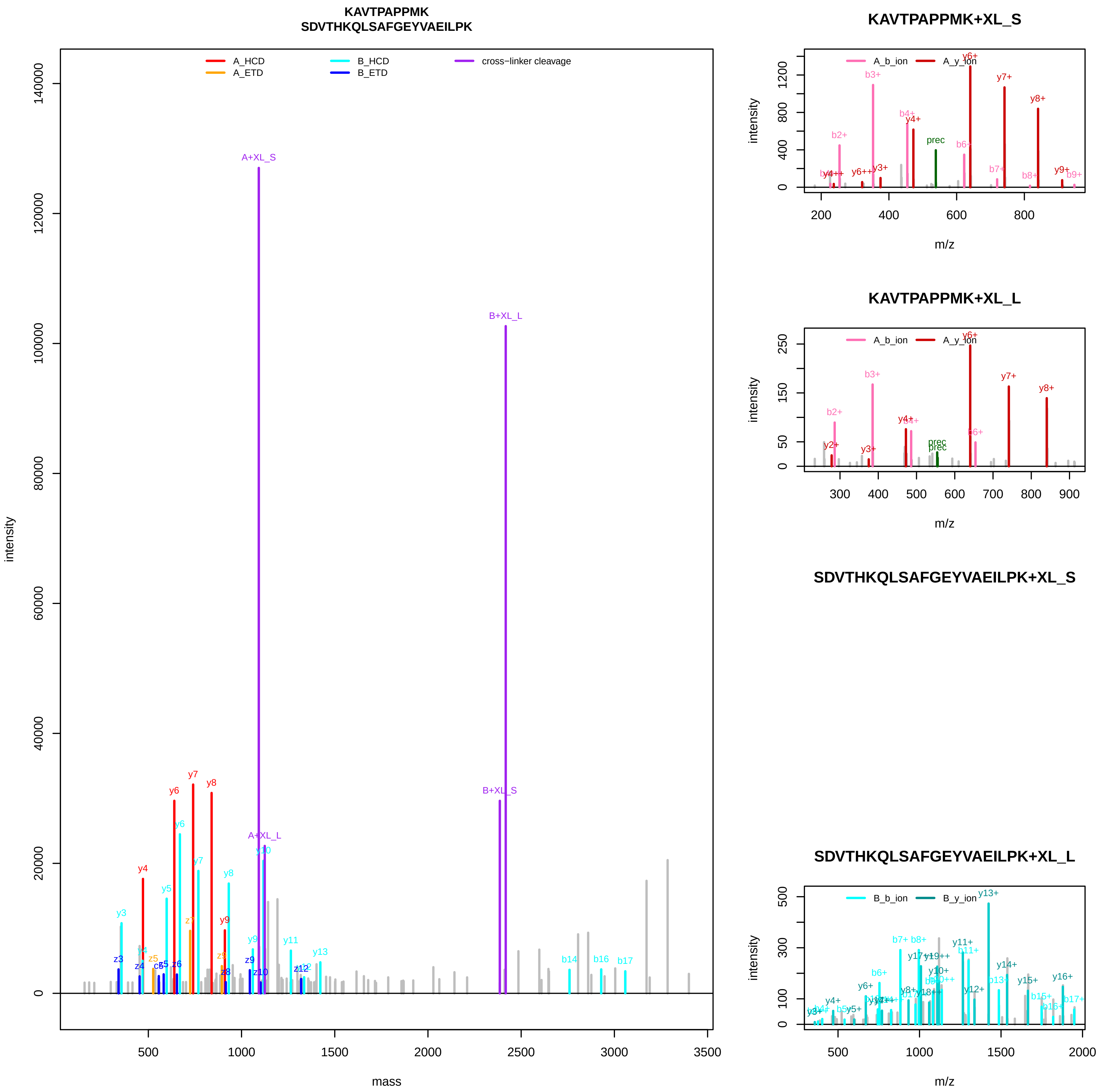
KAVTPAPPMK
SDVTHKQLSAFGEYVAEILPK
A_HCD
A_ETD
B_HCD
B_ETD
cross−linker cleavage
intensity
mass
A+XL_S
B+XL_L
B+XL_S
A+XL_L
y3
y4
y5
y6
y7
y8
y9
y10
y11
y13
z3
z4
z5
z6
z7
z8
z9
z10
z12
b14
b16
b17
KAVTPAPPMK+XL_S
A_b_ion
A_y_ion
b2+
b3+
b4+
b6+
b7+
b8+
b9+
y3+
y4+
y6+
y7+
y8+
y9+
y4++
y6++
prec
m/z
KAVTPAPPMK+XL_L
y2+
b2+
b3+
y3+
y4+
b4+
prec
b6+
y6+
y7+
y8+
SDVTHKQLSAFGEYVAEILPK+XL_S
SDVTHKQLSAFGEYVAEILPK+XL_L
B_b_ion
B_y_ion
y13+
b7+
b8+
y11+
b11+
y19++
y10+
y14+
y12+
b13+
y15+
y16+
b15+
b16+
b17+
y18++
y8+
y6+
y4+
y5+
b5+
b6+
y3+
